# Changes in Registration Parameters for Ongoing Clinical Trials in Ukraine After 2022 Russian Invasion

**DOI:** 10.1001/jamanetworkopen.2023.20202

**Published:** 2023-06-26

**Authors:** Diana Gujinović, Tomislav Viđak, Mariia Melnikova, Nelson Joaquim Fortuna de Sousa, Ana Marušić

**Affiliations:** 1 Department of Pharmacology, University of Split School of Medicine, Split, Croatia; 2 Medical student, University of Split School of Medicine, Split, Croatia; 3 The Regional Health Administration of Lisbon and Tagus Valley, Lisbon, Portugal; 4 Northern Region Health Administration ACES Grane Porto I, Santo Tirso, Portugal; 5 Department of Research in Biomedicine and Health and Center for Evidence-based Medicine, University of Split School of Medicine, Split, Croatia

## Abstract

**Importance:**

Clinical trial activity in Ukraine was seriously affected by the Russian invasion. However, data are lacking on how this conflict affects clinical trials.

**Objective:**

To evaluate whether registered changes to trial information reflect war-related disturbances to trials in Ukraine.

**Design, Setting, and Participants:**

This cross-sectional study included noncompleted trials conducted in Ukraine from February 24, 2022, to February 24, 2023. Trials conducted in Estonia and Slovakia were also analyzed for comparison. Study records within the ClinicalTrials.gov archive were accessed by using the change history feature in the tabular view for each record.

**Exposure:**

Russian invasion of Ukraine.

**Main Outcomes and Measures:**

The frequency of changes to the protocol and results registration parameters before and after the start of the war, February 24, 2022.

**Results:**

A total of 888 ongoing trials conducted only in Ukraine (5.2%) or in multiple countries (94.8%), and enrolling a median of 348 participants were analyzed. Almost all sponsors for 775 industry-funded trials were non-Ukrainian (99.6%). On February 24, 2023, 267 trials (30.1%) were without any recorded update in the registry after the war. In 15 (1.7%) multisite trials, Ukraine was removed as a location country after mean (SD) of 9.4 (3.0) postwar months. The rates of changes in 20 parameters 1 year before and after the war started had a mean (SD) absolute difference of 3.0% (2.5%). Besides study status changes in each study record version, the contacts and locations field was the most frequently modified (56.1%), with a higher rate for multisite (58.2%) than in only Ukrainian (17.4%) trials. This finding was consistent for all analyzed registration parameters. In trials conducted solely in Ukraine, the median number of record versions was the year before (95% CI, 0-0) and after February 2022 (95% CI, 0-1), similar to registered trials from Estonia and Slovakia.

**Conclusions and Relevance:**

Results of this study suggest that war-related changes in trial conduct in Ukraine may not be completely visible in the largest public trial registry, which is expected to present accurate and timely information on clinical trials. These findings raise questions regarding the updating practices for registration information, which should be mandatory, especially in times of crises, to ensure the safety and the rights of trial participants in a war zone.

## Key Points

**Question:**

Was the Russian invasion of Ukraine in 2022 associated with changes in registration data for ongoing clinical trials with Ukrainian sites?

**Findings:**

In this cross-sectional study including 888 trials conducted only in Ukraine or within multiple countries, changes to the registered data in the ClinicalTrials.gov registry 1 year after the beginning of the Russian invasion were mostly similar to those posted 1 year before the war. In trials conducted solely in Ukraine, the number of changes to the trial records was similar before and after the start of the war, and similar to registered trials from Estonia and Slovakia.

**Meaning:**

These findings suggest that trial sponsors may have not been providing war-related changes in trial conduct in Ukraine in the largest public trial registry, expected to present accurate and timely information on clinical trials.

## Teaser

This cross-sectional study evaluates whether the documented war-related disturbances in Ukraine were reflected in the change of registration data elements archived in ClinicalTrials.gov.

## Introduction

On February 24, 2022, Russia launched its invasion of neighboring Ukraine, turning this country into a war site.^1,2^ The war chaos and disruption were also reflected in the health care, including the care for vulnerable populations^3^ and patients with cancer.^4^ Clinical trials were especially adversely affected because of their complexity and integration within multicentric multinational trials.^1^ This led to changes and protocol deviations in the conduct of clinical trials,^1,2^ which may have lasting consequences in terms of delayed diagnoses and treatments and increased mortality, especially from cancer.^5^ In February 2022, the European Medicines Agency advised sponsors to adapt the approaches developed during the COVID-19 pandemic for addressing the war in Ukraine and clinical trials.^6,7^

It has been recently estimated that more than 500 oncology trials in Ukraine and Russia will be affected by war and may cause underpowering of multinational phase 3 trial results.^8^ However, there is no evidence of how the war has actually affected clinical trials. In view of the legal and professional obligations of trial researchers and sponsors,^9^ all changes to the information about the trial protocol have to be publicly available.

With regard to ClinicalTrials.gov, the largest clinical trial registry,^10^ the study sponsor is mostly responsible for submitting, updating, and verifying study information.^11,12^ The changes to the trial record are typically available within 2 to 5 working days.^13^ In times of war, a reliable public record of changes and protocol deviations would be expected, especially if the sponsor is situated outside of Ukraine. The recording of changes is important not only for the scientific community but also for prospective trial participants because it provides easy and rapid access to important information regarding study recruitment.^14^ In this study, we evaluated whether the documented war-related disturbances in Ukraine were reflected in the change of registration data elements archived in ClinicalTrials.gov.

## Methods

### Study Design and Setting

This cross-sectional study used a publicly accessible data set. Institutional review board approval and informed consent were therefore not required. This study followed the Strengthening the Reporting of Observational Studies in Epidemiology (STROBE) reporting guideline for cross-sectional studies.

We first retrieved registration information on July 25, 2022, and included trials if they had (1) a National Clinical Trial identifier, (2) the last update posted before July 25, 2022, (3) Ukraine listed among location countries under the recruitment information section, and (4) any noncompleted recruitment status, including *not yet recruiting*, *recruiting*, *enrolling by invitation*, *active, not recruiting*, *suspended*, *terminated*, *withdrawn* or *unknown*. We did not set any restrictions regarding the first posted date. All trials identified by the initial search were included in the analysis, implying that no additional exclusion criteria were applied. In our second data extraction, on May 1, 2023, we expanded our study observation period to February 24, 2023. Changes in registration data were evaluated regarding 3 time periods: (1) from the first version date to February 24, 2022, the start of the Russian invasion,^15^ (2) 1 year before, and (3) 1 year after this date.

In the second data extraction, we identified trials conducted in Slovakia and Estonia, countries neighboring Ukraine or Russia. Similar inclusion criteria were used, except the last update posted was set to February 24, 2023. We also contacted trialists in Ukraine about their experiences.

### Outcomes

Earlier versions of study records within the ClinicalTrials.gov archive were accessed by using the change history feature in the tabular view for each record.^12,16^ For each trial conducted in Ukraine, we collected information on the total number of study record versions and the number of study record versions in which a particular registration parameter, recorded at least once in the archive field, was changed. The analysis did not include the earliest archived version of the study record, which had no modifications. We collected the information for all registration parameters—data elements, including those required by the International Committee of Medical Journal Editors and World Health Organization International Clinical Trials Registry Platform.^17^ On May 1, 2023, we reevaluated registered data within the recruitment status field for all included trials, as well as the results reporting in the ClinicalTrials.gov results database. For all included trials with sites in Ukraine, we also recorded the date of the first and the last study record version identified in the ClinicalTrials.gov archive field up to February 24, 2023. The date of the first version was used to calculate the median time span for archived changes considering the start of the war as the end date.

For each trial conducted in Estonia or Slovakia, we collected only the information on the total number of study record versions in the ClinicalTrials.gov archive site for equal time periods as for the analysis of Ukrainian trials. We also noted the date of the first and the last archived study record version within the complete observation period.

### Data Extraction

The data were downloaded from ClinicalTrials.gov via comma-separated values export. To test the spreadsheet and reach a consensus on the extraction protocol, study record versions from the ClinicalTrials.gov archive site were reviewed for changes independently by 4 researchers (D.G., T.V., M.M., and N.J.F.S.) for a 10% random sample of the cohort of trials. After confirmation by an independent researcher not involved in data extraction (A.M.), all 4 researchers then carried out independent extraction of the full data set.

### Statistical Analysis

Descriptive analyses were performed using MedCalc version 20.218 (MedCalc Software). Data were presented as frequencies and medians with 95% CIs and IQRs or as a mean (SD) after assessment of the normality of the distribution of data with the D’Agostino-Pearson test.

## Results

On July 25, 2022, using ClinicalTrials.gov advanced search, we identified a total of 888 trials conducted in Ukraine and registered with any recruitment status other than completed. Ukraine was recorded as a single (5.2%) or one of the multicountry sites (94.8%).

Among 855 interventional trials (96.3%), phase 2 (22.0%) and 3 (61.9%) were most common (Table 1). The median primary completion date was 2022 (95% CI, 2022-2022 [IQR, 2019-2023]) for 879 trials with available data, whereas the median completion date was 2023 (95% CI, 2022-2023 [IQR, 2020-2024]) for 881 trials with available data. Trials were mostly funded by the pharmaceutical industry, without other organizations (n = 775 [87.3%]) or with National Institutes of Health, US federal government, and those classified as other (n = 46 [5.2%]). Among sponsors for 775 industry-funded trials, 99.6% originated outside of Ukraine. Of 842 multisite trials, 768 (91.2%) were funded solely by industry, based predominantly outside of Ukraine (99.9%), with only 1 trial sponsored by a Ukrainian company. A total of 46 trials conducted only in Ukraine were funded by organizations categorized as other (63.0%), and only 15.2% by industry only. Among these trials, Ukrainian companies sponsored only 2. Most trials were without registered results until May 1, 2023 (73.4%) (Table 1). A total of 39 trials (4.4%), all with multiple sites, that were without any results on July 25, 2022, posted or submitted their trial findings up to May 1, 2023. With regard to all analyzed trials, the median enrollment was 348 participants (95% CI, 300-379 [IQR, 136-750]) (eTable 1 in Supplement 1). Five months after the start of the war, the recruitment status was recruiting (36.6%) or active, not recruiting (29.3%) for more than half of the trials. At 1 year, 113 of 325 trials (34.8%) with recruiting status on July 25, 2022, changed their status, mostly to active, not recruiting (68.1%), followed by completed (17.7%), terminated (8.8%), unknown (4.4%), or suspended (0.9%) (eTable 2 in Supplement 1). A total of 36 of 46 trials (78.3%) conducted solely in Ukraine did not have any recorded change in the recruitment status on May 1, 2023, and most had unknown status (41.7%). Of all 888 analyzed trials, 90 trials (10.1%) were identified with the status completed during our second extraction, despite the current war situation, and almost all were multicountry trials (93.3%).

**Table 1.** General Characteristics of 888 Trials Having Ukraine as the Only or One of Multiple Sites^a^

Trial characteristic	Trials, No. (%)
Study type	
Observational	33 (3.7)
Interventional	855 (96.3)
Interventional study model^b^	
Single group	124 (14.5)
Sequential	27 (3.2)
Parallel	691 (80.8)
Crossover	9 (1.1)
Factorial	1 (0.1)
Not provided	3 (0.4)
Study phase^b^	
Early phase 1 or phase 1	31 (3.6)
Phase 1/2	25 (2.9)
Phase 2	188 (22.0)
Phase 2/3	30 (3.5)
Phase 3	529 (61.9)
Phase 4	24 (2.8)
Stated not applicable	28 (3.3)
Observational study model^c^	
Cohort	19 (57.6)
Case-only	6 (18.2)
Case-control	3 (9.1)
Family-based	1 (3.0)
Other or not provided	4 (12.1)
Funder	
Industry	775 (87.3)
Industry with other organizations	46 (5.2)
Funders classified as other	61 (6.9)
NIH, alone or with other funders	5 (0.6)
US federal government with other funders	1 (0.1)
First posted date	
Provided^d^	888 (100.0)
Not provided	0
Start date	
Provided^e^	888 (100.0)
Not provided	0
Primary completion date^f^	
Provided^g^	879 (99.0)
Not provided	9 (1.0)
Completion date^h^	
Provided^i^	881 (99.2)
Not provided	7 (0.8)
The first archived study record version date	
Provided^j^	873 (98.3)
Not provided^k^	15 (1.7)
The last archived study record version date^a^	
Provided^l^	873 (98.3)
Not provided^k^	15 (1.7)
Study results^a^	
Posted	230 (25.9)
Submitted	6 (0.7)
Not provided	652 (73.4)

Abbreviation: NIH, National Institutes of Health.

^a^ Data retrieved from ClinicalTrials.gov on July 25, 2022; except the ClinicalTrials.gov Study Results Table of the study record, which was reviewed again on May 1, 2023, and the last study record version identified up to February 24, 2023 (second extraction).

^b^ Related only to interventional study design (n = 855).

^c^ Related only to observational study design (n = 33).

^d^ Median, 2018 (95% CI, 2018-2018 [IQR, 2015-2020]; range, 2002-2022).

^e^ Median, 2018 (95% CI, 2018-2019 [IQR, 2015-2020]; range, 1994-2022).

^f^ According to the ClinicalTrials.gov Glossary of Common Terms: “the date on which the last participant in a clinical study was examined or received an intervention to collect final data for the primary outcome measure.”

^g^ Median, 2022 (95% CI, 2022-2022 [IQR, 2019-2023]; range, 2006-2032).

^h^ According to the ClinicalTrials.gov Glossary of Common Terms: “the date on which the last participant in a clinical study was examined or received an intervention to collect final data for the primary and secondary outcome measures, and adverse events (that is, the last participant’s last visit).”

^i^ Median, 2023 (95% CI, 2022-2023 [IQR, 2020-2024]; range, 2006-2034).

^j^ Median, 2018 (95% CI, 2018-2018 [IQR, 2015-2020]; range, 2005-2022).

^k^ A total of 15 of 888 trials were identified with no changes posted within the tracking information section and thus not included in the analysis.

^l^ Median, 2022 (95% CI, 2022-2022 [IQR, 2021-2023]; range, 2007-2023).

A comparison of the period from the initial registration to February 24, 2022, with the period after the war started until July 25, 2022, found a lower rate of trials with archived changes to registration items after February 24, 2022 (58.1%) than before this date (96.5%). This was also illustrated by the higher median number of study record versions in the ClinicalTrials.gov archive site before the war (19, 95% CI, 17-20 [IQR, 8-38]) in comparison with the median number after (1, 95% CI, 1-1 [IQR, 0-4]) (Table 2). However, the median time span from the initial version to the start of the war was 42 months (95% CI, 39-46 [IQR, 19-81.5] months). If we consider the period of 1 year before the onset of war and 1 year after, almost equal rates of trials with recorded alterations were identified before (70.8%) and after (69.9%), with the median number of study record versions 1 year before the onset of war of 3 (95% CI, 2-3 [IQR, 0-9.5]) and 1 year after of 2 (95% CI, 2-3 [IQR, 0-8]).

**Table 2.** Changes in Registration Parameters for all 888 Trials Conducted in Ukraine Before and After the Start of the Russian Invasion^a^

Registration item	Changes in observation period, No. (%)^b^			
	Before the war in Ukraine		After the war started	
	First version, February 24, 2022^c^	February 24, 2021-February 24, 2022^d^	February 24, 2022-July 25, 2022^e^	February 24, 2022-February 24, 2023^f^
Study identification	490 (55.2)	132 (14.9)	47 (5.3)	85 (9.6)
Study status^g^	857 (96.5)	629 (70.8)	516 (58.1)	621 (69.9)
Recruitment status	791 (89.1)	242 (27.3)	78 (8.8)	223 (25.1)
Sponsor/collaborators	215 (24.2)	39 (4.4)	18 (2.0)	30 (3.4)
Oversight	455 (51.2)	85 (9.6)	15 (1.7)	20 (2.3)
Study description	466 (52.5)	165 (18.6)	62 (7.0)	129 (14.5)
Conditions	206 (23.2)	39 (4.4)	15 (1.7)	26 (2.9)
Study design	550 (61.9)	177 (19.9)	113 (12.7)	245 (27.6)
Arms and interventions	458 (51.6)	146 (16.4)	61 (6.9)	108 (12.2)
Outcome measures^h^	590 (66.4)	226 (25.5)	92 (10.4)	195 (22.0)
Eligibility	597 (67.2)	231 (26.0)	95 (10.7)	185 (20.8)
Contacts and locations	838 (94.4)	566 (63.7)	418 (47.1)	498 (56.1)
IPD sharing	206 (23.2)	52 (5.9)	51 (5.7)	75 (8.4)
References	193 (21.7)	51 (5.7)	38 (4.3)	67 (7.5)
Document section	80 (9.0)	39 (4.4)	18 (2.0)	46 (5.2)
Results	118 (13.3)	19 (2.1)	11 (1.2)	26 (2.9)
Participant flow	70 (7.9)	12 (1.4)	7 (0.8)	20 (2.3)
Baseline characteristics	76 (8.6)	12 (1.4)	10 (1.1)	20 (2.3)
Adverse events	78 (8.8)	15 (1.7)	11 (1.2)	22 (2.5)
More information	60 (6.8)	5 (0.6)	5 (0.6)	13 (1.5)

Abbreviation: IPD, individual participant data.

^a^ Trials with any noncompleted status on July 25, 2022, were included: not yet recruiting, recruiting, enrolling by invitation, active, not recruiting, suspended, terminated, or withdrawn or unknown.

^b^ Trials with 1 or more study record versions with changed registration item.

^c^ Median number of study record versions, 19 (95% CI, 17-20; IQR, 8-38; range, 0-457).

^d^ Median number of study record versions, 3 (95% CI, 2-3; IQR, 0-9.5; range, 0-78).

^e^ Median number of study record versions, 1 (95% CI, 1-1; IQR, 0-4; range, 0-76).

^f^ Median number of study record versions, 2 (95% CI, 2-3; IQR, 0-8; range, 0-79).

^g^ Because study status also includes the date when the last update was posted, this parameter was changed in all trials with 1 or more study record versions noted in the ClinicalTrials.gov archive site.

^h^ Refers both to protocol and study results information.

One year after the war began, 16 of 20 analyzed registration parameters were updated in ClinicalTrials.gov in fewer than a fourth of all trials. All 20 registration parameters identified in the ClinicalTrials.gov archive site had more modifications in the period before the war, from the initial registration, than after February 24, 2022 (Table 2). The mean (SD) of absolute differences between the rates for each registration item was 32.2% (22.0%) regarding July 25, 2022, and 26.7% (18.3%) regarding February 24, 2023, as the end date. The absolute mean (SD) difference between the rates for each registration item was smaller when comparing the interval of 12 months before and after the start of the Russian invasion (3.0% [2.5%]). In this context, contacts and locations and study design were the items with the highest identified rate difference (7.7%).

Between February 2022 and February 2023, 25.1% of trials introduced at least 1 change in the recruitment status field (Table 2). A total of 20 of all 888 noncompleted trials (2.3%) were terminated within 5 months after the start of the war: 10 because of intervention-related reasons, and 10 because of other reasons (4 because of sponsor/business decision, 2 for the lack of recruitment without any further detail and 1 listing COVID-19 pandemic and Ukraine/Russia conflict, 2 for development/investigation plans, and 1 because of budgetary priorities, COVID-19 restrictions and war in Ukraine). No trial was withdrawn and only one was suspended within the 1 year after the start of the war, indicating “change in WHO recommendations for standard of care (control arm) and war in Ukraine where 2 study sites are located” as underlying reasons (NCT04783727). Among 491 trials with active, not recruiting, enrolling by invitation, not yet recruiting, and recruiting statuses detected on May 1, 2023, 31 trials (6.3%) were without any new registration entry in ClinicalTrials.gov 12 months after the start of the Russian invasion.

The parameters that were the most frequently altered from 2022 to 2023 were protocol-related parameters study status (69.9%) and contacts and locations (56.1%) (Table 2). However, both of these parameters had a higher rate of change 1 year before the war (70.8% study status and 63.7% contacts and locations). It should be emphasized that study status was changed in all study record versions since it also includes the date when the last update was posted (eTable 3 in Supplement 1). A higher rate of changes for contacts and locations was found for trials with multiple sites (n = 490 [58.2%]) (Table 3) than for trials with only Ukrainian sites 1 year after the beginning of the war (n = 8 [17.4%]) (Table 4). Of these 8 trials conducted only in Ukraine, 5 deleted the information on contact persons and recruiting status of sites, 1 deleted only the information on contact persons, 1 deleted the information on contact persons and added new Ukrainian sites, and in another 1 trial several Ukrainian sites changed their status from not recruiting to recruiting. This compares with the last recorded changes before the war started to the contacts and locations registry item for trials with only Ukrainian sites: 16 of 46 (34.8%) had a change; 8 deleted the information on contact persons and locations, 2 changed the postal code and details on contact persons, whereas 6 added new information on contact persons, study officials, and site names, alone or with recruiting status. In our second data extraction, Ukraine was removed as the location country in 15 multisite trials (1.7%), after the mean of 9.4 months (SD 3.0 months) since the war began. Five months from February 24, 2022 those trials were recruiting (73.3%), active, not recruiting (20.0%), or enrolling by invitation (6.7%).

**Table 3.** Changes in Registration Parameters for 842 Trials With Sites in Ukraine and Other Countries Before and After the Start of Russian Invasion^a^

Registration item	Changes in observation period, No. (%)^b^			
	Before the war in Ukraine		After the war started	
	First version, February 24, 2022^c^	February 24, 2021-February 24, 2022^d^	February 24, 2022-July 25, 2022^e^	February 24, 2022-February 24, 2023^f^
Study identification	487 (57.8)	131 (15.6)	46 (5.5)	84 (10.0)
Study status^g^	826 (98.1)	620 (73.6)	505 (60.0)	603 (71.6)
Recruitment status	767 (91.1)	236 (28.0)	75 (8.9)	214 (25.4)
Sponsor/collaborators	204 (24.2)	37 (4.4)	17 (2.0)	29 (3.4)
Oversight	447 (53.1)	84 (10.0)	14 (1.7)	17 (2.0)
Study description	454 (53.9)	163 (19.4)	61 (7.2)	128 (15.2)
Conditions	203 (24.1)	38 (4.5)	15 (1.8)	26 (3.1)
Study design	541 (64.3)	175 (20.8)	110 (13.1)	235 (27.9)
Arms and interventions	450 (53.4)	144 (17.1)	60 (7.1)	106 (12.6)
Outcome measures^h^	578 (68.6)	224 (26.6)	91 (10.8)	193 (22.9)
Eligibility	589 (70.0)	229 (27.2)	94 (11.2)	182 (21.6)
Contacts and locations	822 (97.6)	562 (66.7)	414 (49.2)	490 (58.2)
IPD sharing	205 (24.3)	51 (6.1)	51 (6.1)	75 (8.9)
References	189 (22.4)	51 (6.1)	36 (4.3)	66 (7.8)
Document section	80 (9.5)	39 (4.6)	18 (2.1)	46 (5.5)
Results	118 (14.0)	19 (2.3)	11 (1.3)	26 (3.1)
Participant flow	70 (8.3)	12 (1.4)	7 (0.8)	20 (2.4)
Baseline characteristics	76 (9.0)	12 (1.4)	10 (1.2)	20 (2.4)
Adverse events	78 (9.3)	15 (1.8)	11 (1.3)	22 (2.6)
More information	60 (7.1)	5 (0.6)	5 (0.6)	13 (1.5)

Abbreviation: IPD, individual participant data.

^a^ Trials with any noncompleted status on July 25, 2022, were included: not yet recruiting, recruiting, enrolling by invitation, active, not recruiting, suspended, terminated, or withdrawn or unknown.

^b^ Trials with 1 or more study record versions with changed registration item.

^c^ Median number of study record versions, 19.5 (95% CI, 18-21; IQR, 9-40; range, 0-457).

^d^ Median number of study record versions, 3 (95% CI, 2-4; IQR, 0-10; range, 0-78).

^e^ Median number of study record versions, 1 (95% CI, 1-1; IQR, 0-4; range, 0-76).

^f^ Median number of study record versions, 2.5 (95% CI, 2-3; IQR, 0-8; range, 0-79).

^g^ Because study status also includes the date when the last update was posted, this parameter was changed in all trials with 1 or more study record versions noted in the ClinicalTrials.gov archive site.

^h^ Refers both to protocol and study results information.

**Table 4.** Changes in Registration Parameters for 46 Trials With Sites Only in Ukraine Before and After the Start of Russian Invasion^a^

Registration item	Changes in observation period, No. (%)^b^			
	Before the war in Ukraine		After the war started	
	First version, February 24, 2022^c^	February 24, 2021-February 24, 2022^d^	February 24, 2022-July 25, 2022^e^	February 24, 2022-February 24, 2023^f^
Study identification	3 (6.5)	1 (2.2)	1 (2.2)	1 (2.2)
Study status^g^	31 (67.4)	9 (19.6)	11 (23.9)	18 (39.1)
Recruitment status	24 (52.2)	6 (13.0)	3 (6.5)	9 (19.6)
Sponsor/collaborators	11 (23.9)	2 (4.3)	1 (2.2)	1 (2.2)
Oversight	8 (17.4)	1 (2.2)	1 (2.2)	3 (6.5)
Study description	12 (26.1)	2 (4.3)	1 (2.2)	1 (2.2)
Conditions	3 (6.5)	1 (2.2)	0	0
Study design	9 (19.6)	2 (4.3)	3 (6.5)	10 (21.7)
Arms and interventions	8 (17.4)	2 (4.3)	1 (2.2)	2 (4.3)
Outcome measures^h^	12 (26.1)	2 (4.3)	1 (2.2)	2 (4.3)
Eligibility	8 (17.4)	2 (4.3)	1 (2.2)	3 (6.5)
Contacts and locations	16 (34.8)	4 (8.7)	4 (8.7)	8 (17.4)
IPD sharing	1 (2.2)	1 (2.2)	0	0
References	4 (8.7)	0	2 (4.3)	1 (2.2)
Document section	0	0	0	0
Results	0	0	0	0
Participant flow	0	0	0	0
Baseline characteristics	0	0	0	0
Adverse events	0	0	0	0
More information	0	0	0	0

Abbreviation: IPD, individual participant data.

^a^ Trials with any noncompleted status on July 25, 2022, were included: not yet recruiting, recruiting, enrolling by invitation, active, not recruiting, suspended, terminated, or withdrawn or unknown.

^b^ Trials with 1 or more study record versions with changed registration item.

^c^ Median number of study record versions, 1 (95% CI, 1-2; IQR, 0-2; range, 0-47).

^d^ Median number of study record versions, 0 (95% CI, 0-0; IQR, 0-0; range, 0-3).

^e^ Median number of study record versions, 0 (95% CI, 0-0; IQR, 0-0; range, 0-1).

^f^ Median number of study record versions, 0 (95% CI, 0-1; IQR, 0-1; range, 0-2).

^g^ Because study status also includes the date when the last update was posted, this parameter was changed in all trials with 1 or more study record versions noted in the ClinicalTrials.gov archive site.

^h^ Refers both to protocol and study results information.

The trend of a higher rate of changes for multisite trials was also identified for all other protocol-related and results-related parameters with changes archived 12 months after the beginning of the war. The median number of study record versions for 46 trials with sites only in Ukraine was 0 one year before (95% CI, 0-0 [IQR, 0-0]) and 1 year after the start of the Russian invasion (95% CI, 0-1 [IQR, 0-1]) (Table 4). This is in line with the median number of 0 recorded when evaluating registration changes for equal time intervals in trials conducted in Estonia (95% CI, 0-1 [IQR, 0-2] for both periods) and Slovakia (95% CI, 0-0 [IQR, 0-2] for both periods) (Table 5), which were not affected by the war. However, a higher median number of study record versions was identified for 842 trials with sites not only in Ukraine but also in other countries (3 prewar, 95% CI, 2-4 [IQR, 0-10] vs 2.5 postwar, 95% CI, 2-3 [IQR, 0-8]).

**Table 5.** Changes in Registration Parameters for Trials With Sites in Estonia and Slovakia Before and After the Start of Russian Invasion in Ukraine^a^

Variable	Measurement			
	Median	95% CI	IQR	Range
**Date of posting a particular study record, y**				
All trials conducted in Estonia (n = 234)				
The first version in the archive (year)	2017	2016-2017	2012-2019	2005-2022
The last version in the archive (year)	2022	2021-2022	2017-2022	2006-2023
All trials conducted in Slovakia (n = 416)				
The first version in the archive (year)	2016	2015-2017	2010-2019	2005-2023
The last version in the archive (year)	2021	2020-2021	2017-2022	2007-2023
**Observation period, No. of study record versions**				
All trials conducted in Estonia (n = 234)				
Complete				
First version, February 24, 2023	16	13.5-18.5	4-29	0-182
Before the war in Ukraine				
First version, February 24, 2022	14	11-17	3-26	0-182
February 24, 2021-February 24, 2022	0	0-1	0-2	0-35
After the war started				
February 24, 2022-February 24, 2023	0	0-1	0-2	0-21
All trials conducted in Slovakia (n = 416)				
Complete				
First version, February 24, 2023	16	14-18	5-35	0-159
Before the war in Ukraine				
First version, February 24, 2022	15	13-17	3-33	0-159
February 24, 2021-February 24, 2022	0	0-0	0-2	0-40
After the war started				
February 24, 2022-February 24, 2023	0	0-0	0-2	0-30

^a^ Trials with any noncompleted status on February 24, 2023, were included: not yet recruiting, recruiting, enrolling by invitation, active, not recruiting, suspended, terminated, or withdrawn or unknown.

## Discussion

Results of our study suggest that clinical trial registries, such as ClinicalTrials.gov, may not be considered a completely reliable source to provide a deeper understanding of what is happening with the trials conducted during the war in Ukraine, as 30% of 888 trials analyzed in our study were without any archived alteration of registration content after the start of the Russian invasion.

Despite the warning of the European Medicines Agency that “certain changes and protocol deviations are unavoidable” in specific circumstances during the ongoing war in Ukraine,^18^ and that “substantial changes should be appropriately documented,”^6^ we found that 16 of 20 analyzed protocol-related and results-related registration parameters were updated in ClinicalTrials.gov after the war began in fewer than 25% of all trials. When interpreting this finding, several factors should be kept in mind. Within 12 months from the start of the war, international trials with multiple sites in general had a higher rate of updates of registration content in comparison with only Ukrainian trials (72% vs 39%). Despite multisite trials having greater complexity in contrast to single-country studies,^19^ it seems that greater coordination and control, at least that related to registration update, was a possible advantage of these trials in war circumstances. Still, it is challenging to discuss whether a higher rate of updates was expected for both multination and single-nation trials. It should be kept in mind that 91.2% of 842 multisite trials were funded solely by industry, among which only 1 trial was sponsored by a Ukrainian company, and all of the remaining trials by companies located outside of Ukraine and the current war situation. In contrast, only 15.2% of 46 trials conducted solely in Ukraine were funded only by industry, of which 2 trials were sponsored by Ukraine-based companies. Multisite trials in comparison with only Ukrainian trials also had a higher rate of completion (93% vs 7%), considering 90 trials with completed status identified on May 1, 2023.

Theoretically, war is an emergency reason for updating information of an ongoing trial, especially the information important to trial participants, including any tracking, descriptive, recruitment, or administrative information for which participants could search in a public trial registry. As an item significant for recruitment, contacts and locations was the most frequently changed with an intention in all analyzed groups of trials, several-fold more than other registration parameters. However, keeping in mind that war could have direct or indirect outcomes on many trial elements^6^ and that trials included in our study were in different phases, it is challenging to precisely hypothesize which particular registration item is expected to be updated more in this unprecedented situation in comparison with others. Considering the comparison of registration changes identified for the annual intervals in 2021-2022 and in 2022-2023, it is difficult to explain why contacts and locations had more changes before than after the start of the war (63.7% vs 56.1%). Also, it is difficult to explain why study design is an item with the same mean prewar – postwar rate difference as contacts and locations, but with more changes after the war began.

Regarding results-related registration information, it should be kept in mind that only 26% of trials had registered summarized trial findings until our last extraction date, which could partially explain low rates of any updates, such as participant flow, baseline characteristics, adverse events, outcome measures, and more information. Moreover, the median primary completion date for most trials was 2022. According to Section 801 of the US Food and Drug Administration Amendments Act of 2007,^20^ results information must be submitted by the responsible party no later than 1 year after the primary completion date. For several results-related items, this could be a potential source of bias in our study. However, even before the final submission of trial findings, any interference related to data collection, analysis, and interpretation of findings should ideally be stored in the trial registry, to increase the trial transparency as much as possible, especially in times of war.

Our findings do not exclude the possibility that ClinicalTrials.gov does not appropriately reflect real-life trial adaptations with regard to the war in Ukraine. We contacted colleague trialists from Ukraine, who told us that the situation with clinical trials improved in the first months of 2023, with increased security, logistics, and infrastructure, especially in the central and western regions, as well as regulatory support from the Ministry of Health. However, they reported the lack of new clinical trials and attrition of active trials. Most trials with already enrolled patients resumed soon after the start of the war. In the vast majority of trials with active enrollment, the enrollment was stopped, and almost all trials that were ready to be initiated did not start. Despite some sponsors deciding to terminate particular trial protocols, this was not the common practice. In some trials, recruitment restarted several months after the invasion, and currently there are 16 trials with open enrollment, according to the information we received. Two trials sponsored by Ukrainian companies, with recruiting and unknown status still in ClinicalTrials.gov, are currently reported as completed in the Ukrainian registry of clinical trials. Trial participants were informed about possible changes to the protocol only through sites and investigative teams; direct contact with patients and public platforms were not used. Although the FDA regulatory guidance^21^ and European Medicines Agency 2020 regulatory guidance^7^ were published to direct the handling of trial-related disruptions due to the COVID-19 pandemic, it seems that they were insufficient to address the war-related challenges. Although it has been proposed to use the war analogy for the conduct of clinical trials in the COVID-19 pandemic,^22^ it is equally possible to apply the experience about the effectiveness of recommendations from the pandemic to the new current war situation in Europe.

To protect the rights and safety of study participants and maintain the quality and completeness of reporting,^1,2^ several actions are possible. Investigators and sponsors should use the CONSERVE (CONSORT and SPIRIT Extension for RCTs Revised in Extenuating Circumstances) 2021 guideline, which includes detailed checklists on how to report trial protocols and completed trials altered because of extenuating circumstances.^23^ Also, participants should be informed before their inclusion in a trial to check the information announced in a trial registry on a regular basis, because this is the fastest way to inform all participants in one moment about any change regarding the conduct of a trial. Regulatory agencies should put more focus on the need for a prompt update of trial registration data and develop more circumstance-specific guidance, with a reasonable submission deadline. ClinicalTrials.gov quality control professionals should pay special attention to ongoing trials conducted in a country or countries affected by the war and contact investigators if no updates are posted after a reasonable time. In addition, a new registry item or field could be introduced to address unexpected circumstances requiring alteration of the trial protocol.^24^

### Limitations

To our knowledge, this is the first study to provide an overview of changes of registration items in the archive field of ClinicalTrials.gov that occurred for Ukraine’s clinical trials in the postwar period. Use of a single registry could affect the external validity of our study, but ClinicalTrials.gov is the largest clinical trial database.^10^ We did not analyze qualitative changes in each registration parameter, ie, the content of the data provided in a registry field. Our aim was to collect only numerical objective data about the trial status of clinical trials to prevent subjective data interpretation of content changes. We also compared numerical entries of changes introduced within the archive fields after February 24, 2022, with those before this date. It should be kept in mind that the median time span for archived changes regarding the date of the first archived version and February 24, 2022, was 42 months. However, to prevent any possible bias, we also provided data for the observation period of comparable duration: 1 year before the start of the war and 1 year after. We did not include only the trials that were recruiting participants in our analysis—this was done to get a broader understanding of Ukrainian trials that were not completed at different time points before and after the war, considering that this is the only type of study that has ended normally, according to the ClinicalTrials.gov Glossary of Common Site Terms.^25^ It should also be kept in mind that although trial reporting rates increased along with the US Food and Drug Administration Amendments Act of 2007 legislation and International Committee of Medical Journal Editors reporting requirements, many studies still remain without complete, accurate, and timely information entry in trial registries.^12^ We used ClinicalTrials.gov in our study since important practices are being implemented in this registry, such as sending reminders about the trial status and having protocol and results quality control review by National Library of Medicine staff.^26,27^ However, earlier records in ClinicalTrials.gov are not subjected to new regulation, policies, and scientific practices.^12^ This could be another source of bias when comparing the observation period before the war, starting from the first record version to the period after the start of the war. To address it, we provided comparable prewar–postwar analysis for 2 war-unrelated countries, Estonia and Slovakia. Finally, results on the registration of trial information cannot be assumed to reflect the quality of care provided to the patients in Ukraine.

## Conclusion

Our study found that 888 ongoing noncompleted trials with sites in Ukraine did not have the expected increase in registration changes after the Russian invasion started on February 24, 2022, in comparison with the changes from the year before. Actions are needed to ensure the transparency of clinical trials emergency situations to protect trial participants in terms of their rights and health needs, keep accurate and undistorted trial records, and preserve the ethical and scientific integrity of clinical trials.

## Notes

Supplement 1. **eTable 1.** Characteristics of Participants’ Recruitment in 888 *ClinicalTrials.gov* Trials Having Ukraine as the Only or One of Multiple Sites on 25 July 2022 **eTable 2.** Changes in The Recruitment Status Until 1 May 2023 Identified in 203 *ClinicalTrials.gov* Trials Conducted in Ukraine **eTable 3.** Sub-Parameters Referring to a Particular Registration Parameter Within the *ClinicalTrials.gov* Archive Fields

Supplement 2.
Data Sharing Statement
