# Supplementary material for: Changes in Registration Parameters for Ongoing Clinical Trials in Ukraine After 2022 Russian Invasion
Source: JAMA Netw Open. 2023 Jun 26;6(6):e2320202. doi: 10.1001/jamanetworkopen.2023.20202 (PMC10293907; doi:10.1001/jamanetworkopen.2023.20202)
Supplement: Supplement 1. — eTable 1. Characteristics of Participants’ Recruitment in 888 ClinicalTrials.gov Trials Having Ukraine as the Only or One of Multiple Sites on 25 July 2022 eTable 2. Changes in The Recruitment Status Until 1 May 2023 Identified in 203 ClinicalTrials.gov Trials Conducted in Ukraine eTable 3. Sub-Parameters Referring to a Particular Registration Parameter Within the ClinicalTrials.gov Archive Fields [file jamanetwopen-e2320202-s001.pdf]

Supplemental Online Content

Gujinović D, Viđak T, Melnikova M, Fortuna de Sousa NJ, Marušić A. Changes in registration parameters for ongoing clinical trials in Ukraine after 2022 Russian invasion. *JAMA Netw Open.* 2023;6(6):e2320202. doi:10.1001/jamanetworkopen.2023.20202

eTable 1. Characteristics of Participants’ Recruitment in 888 *ClinicalTrials.gov* Trials Having Ukraine as the Only or One of Multiple Sites on 25 July 2022

eTable 2. Changes in The Recruitment Status Until 1 May 2023 Identified in 203 *ClinicalTrials.gov* Trials Conducted in Ukraine

eTable 3. Sub-Parameters Referring to a Particular Registration Parameter Within the *ClinicalTrials.gov* Archive Fields

This supplemental material has been provided by the authors to give readers additional information about their work.

© 2023 Gujinović D et al. *JAMA Network Open*.

eTable 1. Characteristics of participants' recruitment in 888 *ClinicalTrials.gov* trials having Ukraine as the only or one of multiple sites on 25 July 2022

Trial characteristics	No. (%) of trials
**Recruitment Status:[a]**	
Active, not recruiting	260 (29.3)
Enrolling by invitation	15 (1.7)
Not yet recruiting	3 (0.3)
Recruiting	325 (36.6)
Suspended	3 (0.3)
Terminated[b]	232 (26.1)
Unknown	42 (4.7)
Withdrawn	8 (0.9)
**Sample size:**	
Provided ≠ zero (median 348, 95% CI 300-379, IQR: 136-750, range 1-37000)	880 (99.1)
Provided as zero	8 (0.9)
**Gender:**	
Male	25 (2.8)
Female	51 (5.7)
Both	811 (91.3)
Not provided	1 (0.1)
**Minimum age:**	
Provided (median 18, 95% CI: 18-18, IQR: 18-18, range: 1 minute-65 years)	857 (96.5)
Not provided	31 (3.5)
**Maximum age:**	
Provided (median 74, 95% CI: 68.3-75, IQR: 55-80, range: 48 hours-130 years)	404 (45.5)
Not provided	484 (54.5)
**Age group:**	
Adult	53 (6.0)
Adult/Older adult	677 (76.2)
Child/Adult/Older adult	69 (7.8)
Child	64 (7.2)
Child/Adult	20 (2.3)
Older adult	5 (0.6)

Abbreviations: CI, confidence interval; IQR, interquartile range.
[a] Data retrieved on 1 May 2023: Active, not recruiting (n = 261, 29.4%), Enrolling by invitation (n=12, 1.4%), Not yet recruiting (n=1, 0.1%), Recruiting (n=217, 24.4%), Suspended (n=1, 0.1%), Terminated (n=253, 28.5%), Unknown (n=45, 5.1%), Withdrawn (n= 8, 0.9%) and Completed (n=90, 10.1%).
[b] A total of 77 out of these 232 trials (33.2%) had at least one change a year before the war began until 24 Feb 2022, and 51 (22.0%) at least one change in the registration record between this date and 24 Feb 2023.

© 2023 Gujinović D et al. *JAMA Network Open*.

eTable 2. Changes in the recruitment status until 1 May 2023 identified in 203 *ClinicalTrials.gov* trials conducted in Ukraine

Recruitment status on July 25, 2022	Recruitment status on May 1, 2023	No. (%) of trials
Active, not recruiting	Completed	61 (30.0)
Recruiting	Completed	20 (9.9)
Unknown	Completed	4 (2.0)
Enrolling by invitation	Completed	2 (1.0)
Not yet recruiting	Completed	1 (0.5)
Terminated	Completed	1 (0.5)
Suspended	Completed	1 (0.5)
Recruiting	Active, not recruiting	77 (37.9)
Enrolling by invitation	Active, not recruiting	1 (0.5)
Active, not recruiting	Recruiting	4 (2.0)
Suspended	Recruiting	1 (0.5)
Active, not recruiting	Terminated	10 (4.9)
Recruiting	Terminated	10 (4.9)
Not yet recruiting	Terminated	1 (0.5)
Suspended	Terminated	1 (0.5)
Recruiting	Suspended	1 (0.5)
Active, not recruiting	Unknown	2 (1.0)
Recruiting	Unknown	5 (2.5)

© 2023 Gujinović D et al. *JAMA Network Open*.

eTable 3. Sub-parameters referring to a particular registration parameter within the *ClinicalTrials.gov* Archive fields

**Parameters listed under "Complete list of historical versions" available within the CT.gov Tabular View**	**Sub-parameters listed when "Comparison Format" is chosen for two study record versions with different dates listed under "Complete list of historical versions"**
Study Identification	Unique Protocol ID, Brief Title, Official Title, Secondary IDs
Study Status	Record Verification, *Overall Status*, Study Start, Primary Completion, Study Completion, First Submitted, First Submitted that Met QC Criteria, First Posted, Last Update Submitted that Met QC Criteria, Last Update Posted, *Results First Submitted, Results First Submitted that Met QC Criteria, Results First Posted*
Recruitment Status	Sub-parameter *Overall Status* under parameter Study Status
Sponsor/Collaborators	Sponsor, Responsible Party, Collaborators
Oversight	U.S. FDA-regulated Drug, U.S. FDA-regulated Device, Data Monitoring
Study Description	Brief Summary, Detailed Description
Conditions	Conditions, Keywords
Study Design	For interventional trials: Study Type, Primary Purpose, Study Phase, Interventional Study Model, Number of Arms, Masking, Allocation, Enrollment<br>For observational trials: Study Type, Observational Study Model, Time Perspective, Biospecimen Retention, Biospecimen Description, Enrollment, Number of Groups/Cohorts, Target Follow-Up Duration
Arms and Interventions	Arms, Assigned Interventions
Outcome Measures	Primary Outcome Measures, Secondary Outcome Measures, Other Outcome Measures<br>*In the Results Section*[a]*:* Outcome Measure Information, Statistical Analyses, Statistical Analysis Overview, Comparison Group Selection, Type of Statistical Test, Statistical Test of Hypothesis, Method of Estimation, Other Statistical Analysis
Eligibility	Minimum Age, Maximum Age, Sex, Gender Based, Accepts Healthy Volunteers, Criteria (Inclusion and Exclusion)
Contacts and Locations	Central Contact Person, Central Contact Backup, Locations
IPD Sharing	Plan to Share IPD
References	Citations, Links, Available IPD/Information
Document Section	Study Protocol, Statistical Analysis Plan
Results	The first submission of results within the modules Participant Flow, Baseline Characteristics, Outcome

© 2023 Gujinović D et al. *JAMA Network Open.*

	Measures, Adverse Events, Limitations and Caveats, and/or More Information<br>Sub-parameters *Results First Submitted, Results First Submitted that Met QC Criteria, Results First Posted* under parameter Study Status
Participant Flow[a]	Recruitment Details, Pre-assignment Details, Arm/Group Information, Type of Units Assigned
Baseline Characteristics[a]	Arm/Group Information, Baseline Analysis Population Information, Baseline Measure Information
Adverse Events[a]	All-Cause Mortality, Serious Adverse Events, Other (Not Including Serious) Adverse Events
More Information[a]	Certain Agreement, Results Point of Contact

[a]According to the *ClinicalTrials.gov* Results Data Element Definitions for Interventional and Observational Studies.

© 2023 Gujinović D et al. *JAMA Network Open.*
