# Supplementary material for: Changes in Registration Parameters for Ongoing Clinical Trials in Ukraine After 2022 Russian Invasion
Source: JAMA Netw Open. 2023 Jun 26;6(6):e2320202. doi: 10.1001/jamanetworkopen.2023.20202 (PMC10293907; doi:10.1001/jamanetworkopen.2023.20202)
Supplement: Supplement 2. — Data Sharing Statement [file jamanetwopen-e2320202-s002.pdf]

Data Sharing Statement

Gujinović. Changes in Registration Parameters for Ongoing Clinical Trials in Ukraine After 2022 Russian Invasion. *JAMA Netw Open*. Published June 26, 2023. doi:10.1001/jamanetworkopen.2023.20202

Data
Data available: Yes
Data types: Data (not involving human participants)
How to access data: Available upon request from the first author, Dr Diana Gujinovic, diana.gujinovic@mefst.hr. The data will also be posted in OSF after publication
When available: With publication

Supporting Documents
Document types: None

Additional Information
Who can access the data: Anyone requesting the data
Types of analyses: For any purpose
Mechanisms of data availability: Without investigator support
